# Vital Pulp Therapy in Permanent Teeth with Irreversible Pulpitis Caused by Caries: A Prospective Cohort Study

**DOI:** 10.3390/jpm11111125

**Published:** 2021-11-01

**Authors:** Xiaoxu Guan, Yi Zhou, Qingxia Yang, Tianer Zhu, Xuepeng Chen, Shuli Deng, Denghui Zhang

**Affiliations:** Stomatology Hospital, School of Stomatology, Zhejiang University School of Medicine, Zhejiang Provincial Clinical Research Center for Oral Diseases, Key Laboratory of Oral Biomedical Research of Zhejiang Province, Cancer Center of Zhejiang University, Hangzhou 310006, China; 7312020@zju.edu.cn (X.G.); zyuthscsa@zju.edu.cn (Y.Z.); dentist-yang@zju.edu.cn (Q.Y.); zhutianer@zju.edu.cn (T.Z.); cxp1979@zju.edu.cn (X.C.); dengshuli@zju.edu.cn (S.D.)

**Keywords:** vital pulp therapy, irreversible pulpitis, iRoot BP Plus, personalized treatment, caries

## Abstract

Background: When a tooth is diagnosed with irreversible pulpitis, root canal therapy (RCT) is generally performed to completely remove pulp tissue, which might lead to a higher risk of loss of vascularity, and teeth being more prone to fracture. Vital pulp therapy (VPT) is a personalized method of treating irreversible pulpitis, which conforms to the trend of minimally invasive endodontics. The remaining vital pulp could promote the physiological development of the roots of young permanent teeth with incomplete apical foramen. However, clear guidelines for VPT indication are still missing. Objective: This prospective cohort study evaluated the outcomes of vital pulp therapy (VPT) using iRoot BP Plus (Innovative Bioceramix Inc, Vancouver, BC, Canada) in permanent teeth of 6- to 20-year-old patients with irreversible pulpitis caused by caries and analyzed the preoperative factors affecting VPT prognosis. Methods: Fifty-nine permanent teeth in 59 patients with irreversible pulpitis caused by caries were treated with VPT using iRoot BP Plus. All patients received VPT under a standardized protocol. After informed consent, teeth were isolated with a dental dam, then operators performed VPT with iRoot BP Plus and restored the teeth with composite resin or stainless steel crown. Patients were postoperatively recalled after 3, 6 and 12 months and then recalled annually. Successful cases were defined as successful in both clinical and radiographic evaluations. A statistical analysis was performed using the Fisher exact test, and the level of significant difference was *p* < 0.05. Results: After 6–36 months of follow-up, a total of 57 teeth from 57 patients were accessible for evaluation. The mean age of subjects was 11.75 ± 3.81 years. The overall clinical and radiographic success rate of VPT was 91.2% (52/57). With an observation time of one year or more, the success rate was 90.5% (38/42). All the symptoms and physical examination findings showed no significant effect on VPT prognosis (*p* > 0.05) using a binary logistic regression model. Conclusions: Permanent teeth in 6- to 20-year-old patients diagnosed as irreversible pulpitis caused by caries can be successfully treated with VPT using iRoot BP Plus.

## 1. Introduction

In the traditional concept, when pulp inflammation reaches a certain degree, it is diagnosed as irreversible pulpitis, and root canal therapy (RCT) is generally performed to remove pulp tissue completely. However, RCT could cause losses in vascularity and eventually make the tooth more prone to fracture [1]. Although RCT was the standard of care, the long-term preservation rate of offending teeth after RCT was significantly lower than that of vital teeth, and this phenomenon was particularly significant in molars [2]. This could be because vital teeth with more soft and hard tissues have a stronger resistance to occlusal forces within the physiological range [3]. In order to retain more soft and hard tissues compared to RCT, vital pulp therapy (VPT) is considered a promising personalized treatment for irreversible pulpitis by removing a certain amount of pulp, according to pulpal status. VPT conforms to the trend of minimally invasive endodontics. The remaining vital pulp could promote the physiological development of the roots of young permanent teeth with incomplete apical foramen [4,5]. For mature permanent teeth, VPT can also be considered because mature vital pulp tissue had a spontaneous tendency to heal in relatively sterile conditions histologically [6]. The protective effect of vital pulp reduced the risk of root fracture in the offending teeth, so it should be preserved as much as possible in the endodontic treatment of permanent teeth.

The VPT for irreversible pulpitis with carious exposure is divided into direct pulp capping (DPC), a partial pulpotomy (PP) and full pulpotomy (FP) according to the amount of pulp removed. The pulp-capping agent directly covers the exposed pulp during DPC. Part of the coronal pulp is removed during PP, and all the coronal pulp is removed during FP; then, the remaining pulp tissue is covered by a pulp-capping agent. Asgary et al. performed DPC, PP, FP and indirect pulp capping in mature permanent molars with clinical signs of irreversible pulpitis and found that the four VPTs were all associated with successful clinical and radiographic outcomes [7]. However, clear guidelines for its indication are still missing.

There is a clear distinction between the application of VPT as a treatment approach to immature or mature teeth diagnosed with irreversible pulpitis. For the former (immature), this is a widely accepted treatment, while the evidence to support this treatment for mature teeth is still scarce and under discussion as a possible alternative option to the conventional RCT. A recent systematic review appraised the current best evidence for the application of VPT in mature teeth with irreversible pulpitis and found favorable outcomes for PP and FP. However, no study with DPC was able to meet the inclusion criteria, which shows that research is still needed in this field [8].

The ideal pulp-capping agents should have nontoxic, antibacterial, anti-inflammatory and good sealing properties and should have the ability to induce dentin mineralization [9]. iRoot BP Plus (Innovative Bioceramics, Vancouver, BC, Canada) is a relatively new bioceramic material, which has shown similar results to Mineral Trioxide Aggregate (MTA) in the pulpotomy of dog teeth [10] and has a better clinical handling performance than MTA. Research has shown that iRoot BP Plus possessed good biocompatibility, the ability to induce mineralization and odontoblast differentiation [11]. It is considered a suitable substitute for calcium hydroxide in the pulpotomy of permanent teeth [12]. iRoot BP Plus has good prospects in clinical application as a pulp-capping agent.

This study aimed to evaluate the clinical and radiographic outcomes of VPT using iRoot BP Plus in permanent teeth with irreversible pulpitis caused by caries and analyzed the preoperative factors affecting VPT prognosis.

## 2. Materials and Methods

### 2.1. Ethics Statement

The study protocol was approved by the local Ethics Committee (The Affiliated Hospital of Stomatology, School of Stomatology, Zhejiang University School of Medicine) and registered in the Chinese Trials Registry (No. ChiCTR2100044580). Informed consent was obtained from all patients.

### 2.2. Design and Participants

**Figure 1:**
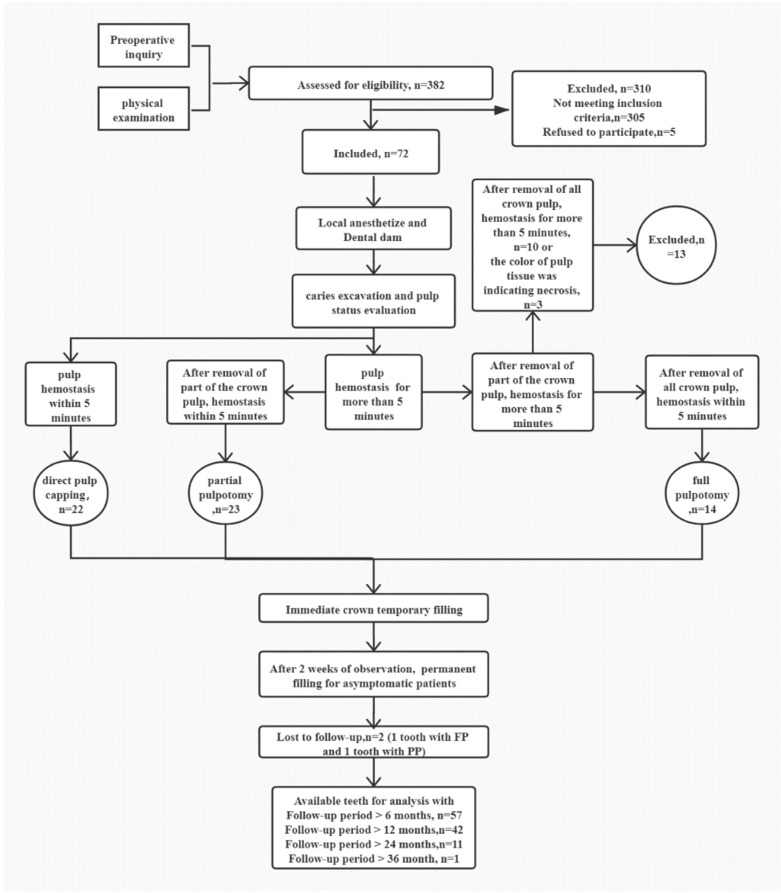
A schematic diagram of the VPT protocol in this clinical study. The number (n) of patients participating in each stage is given. In general, after anesthetizing and isolating the tooth before caries excavation and pulp exposure, pulp bleeding was assessed, and the pulp status was evaluated under microscope. If pulp hemostasis was achieved by direct contact with a cotton pellet moistened with 1% NaOCl within 5 min, DPC was performed. If the hemostasis was more than 5 min, approximately 2–3 mm or more of affected pulpal tissue underneath the exposure site was removed using a sterile high-speed diamond. After this, if the hemostasis was less than 5 min, PP was performed. Otherwise, the full crown pulp was removed to the level of the root canal orifices. Hereafter, if the hemostasis was less than 5 min, FP was performed. Otherwise, the treated tooth was excluded from the study and further treated with RCT or revascularization according to the root development.

The overall protocol was graphically described graphically in Figure 1. The guidelines of Strengthening the Reporting of Observational Studies in Epidemiology (STROBE; https://www.strobe-statement.org/index.php?id=available-checklists, accessed on 1 April 2019) were used to ensure proper reporting of the results. Patients aged from 6 to 20 years attending the Department of Endodontics, The Affiliated Hospital of Stomatology, School of Stomatology, Zhejiang University School of Medicine, were recruited.

### 2.3. Inclusion Criteria

The inclusion and exclusion criteria were set based on the AAE guidelines (https://www.aae.org/specialty/newsletter/endodontic-diagnosis/, accessed on: 1 April 2019) and Levin’s research [13]:

(1) Posterior teeth with deep caries exposing the pulp;
(2) Posterior teeth with preoperative symptoms, such as referred pain, spontaneous pain or pain induced in thermal and cold sensitivity tests, which lasted from several seconds to even hours compared to the control teeth;
(3) Posterior teeth with no prominent radiolucency at the periapical or furcation regions;
(4) In order to ensure the independence of samples and the fitting of statistical models, one tooth was selected from each participant, with multiple teeth receiving VPT for analysis; the priority order was first premolar, second premolar, second molar and first molar.

### 2.4. Exclusion Criteria

(1) Patients with general contraindications to dental treatment;
(2) Teeth with completed RCT or VPT;
(3) Teeth with external or internal resorption, prominent radiolucency at the periapical regions, or furcation;
(4) Teeth with pulpitis caused by wedge-shaped defects, cracking, periodontitis or tooth fracture;
(5) Teeth not responsive to vitality tests.

### 2.5. Interventions

After clinical examination, a periapical radiograph was taken by a periapical film machine (Sirona, Morbach, Germany), and the preoperative diagnosis was established. The verbal numerical rating scale (vNRS) was applied to postoperatively record pain intensity, as the vNRS reliability was strong in children 6 years and older [14].

After clinical and radiographic evaluation, three endodontic postgraduate students performed all operations, following the same protocol and under the supervision of an experienced instructor. Firstly, 4% articaine with 1:100,000 epinephrine (ACTEON, Mérignac, France) was administrated to anesthetize the tooth. Then, a dental dam (Kerr, Iserlohn, Germany) was applied to isolate the tooth before caries excavation using a sterile high-speed diamond under water cooling, 5% sodium hypochlorite (NaOCl) was applied to disinfect the tooth surface, and 2.5% NaOCl was used to rinse the cavity. After pulp exposure, pulp bleeding was assessed, and the pulp status was evaluated. If pulp hemostasis was achieved by direct contact with a cotton pellet moistened with 1% NaOCl within 5 min, DPC was performed. If the hemostasis took longer than 5 min, approximately 2–3 mm or more of affected pulpal tissue was removed underneath the exposure site using a sterile, high-speed diamond. After this, if the hemostasis took less than 5 min, PP was performed. Otherwise, the full-crown pulp was removed to the level of the root canal orifices. Hereafter, if the hemostasis took less than 5 min, FP was performed. Otherwise, the treated tooth was excluded from the study and further treated with RCT or revascularization according to the root development. Subsequently, iRoot BP Plus was prepared according to the manufacturer’s instructions, and a 3-mm layer was placed on the pulp tissue. After iRoot BP Plus was covered with a cotton pellet, resin-modified glass ionomer (Vitrebond, 3M, ESPE, St Paul, MN, USA) was placed over the iRoot BP Plus layer as a protective base. Then, resin composite or a stainless-steel crown was applied to restore the tooth, depending on the amount of tooth structure left.

### 2.6. Outcomes

The patients were asked to undergo a clinical and radiographic examination after 3, 6 and 12 months, and annually thereafter. The case was regarded as successful if: (1) there was no history of spontaneous discomfort or pain; (2) there was no tenderness to percussion, palpation or cold and heat stimuli; (3) the mobility was not higher than grade I; (4) there were normal soft tissues around the tooth with no swelling or sinus tract; (5) the filling materials were intact, and the function was normal; (6) there was no periradicular tissue pathosis, and no radiographic external resorption; (7) there was no intraradicular pathosis, and no radiographic internal resorption; (8) there was continued radiographic root development in immature roots.

### 2.7. Statistical Analysis

A binary logistic regression model was used to compare the outcomes between different baseline characteristics and VPT types; significance was set at *p* < 0.05.

## 3. Results

The results of the Cohen Kappa statistics showed good intraobserver and interobserver agreement. The observers scored within the 0.75–0.85 range for reliability.

Fifty-nine permanent teeth in 59 patients with irreversible pulpitis caused by caries were treated with VPT using iRoot BP Plus. Fifty-seven participants (27 males and 30 females, 6–20 years old) with 57 permanent teeth were accessible for evaluation, and the follow-up rate was 96.6%. The mean age of patients receiving VPT was 11.75 ± 3.81 years. The follow-up examination period ranged from 6 to 36 months, with a mean of 16.0 ± 6.58 months.

**Figure 2:**
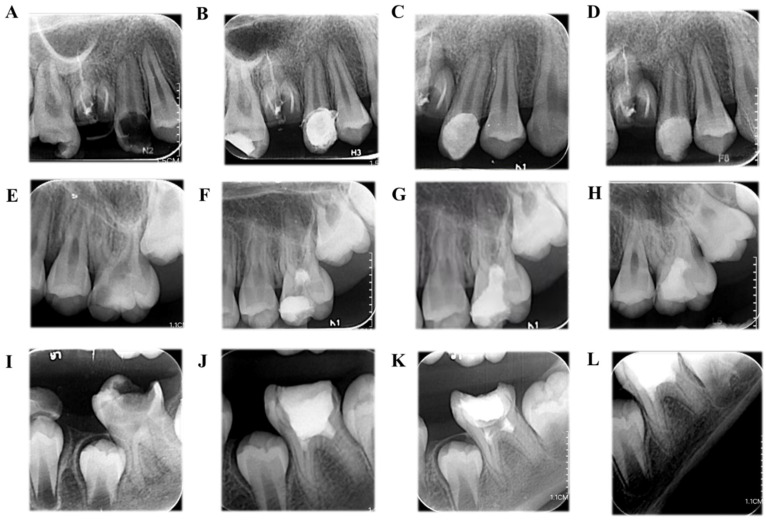
(**A**) The preoperative periapical radiograph of the upper-right second premolar in an 18-year-old male patient with irreversible pulpitis, clinical symptoms and mature roots. (**B**) The postoperative periapical radiograph after direct pulp-capping (DPC) with iRoot BP Plus. (**C**) The 12-month follow-up. (**D**) The 24-month follow-up (**E**) The preoperative periapical radiograph of the upper left first molar in a 13-year-old male patient with irreversible pulpitis of clinical symptoms and mature roots. (**F**) The postoperative periapical radiograph after partial pulpotomy (PP) with iRoot BP Plus. (**G**) The 6-month follow-up. (**H**) The 12-month follow-up. (**I**) The preoperative periapical radiograph of the lower right first molar in a 9-year-old female patient with irreversible pulpitis of clinical symptoms and immature roots. (**J**) The postoperative periapical radiograph after full pulpotomy (FP) with iRoot BP Plus. (**K**) The 12-month follow-up. (**L**) The 24-month follow-up showing continued root development.

During the follow-up periods, the success rate of VPT was 91.2% (52/57). In all cases, the success rate of DPC was 95.4% (21/22), PP was 90.9% (20/22) and FP was 84.6% (11/13). The radiographs of typical cases of DPC, PP and FP are presented in Figure 2.

The SPSS 25.0 software was applied to construct a binary logistic regression model, and logistic regression analysis was performed on the prognosis and its influencing factors. Taking prognosis as the dependent variable and selecting many factors in Table 1 as independent variables, a binary logistic regression model was fitted. According to the model structure, the independent variables are sorted and summarized, and the values are assigned according to the law.

Before fitting, the regression coefficient variance decomposition method (RVCD) was used to diagnose the multicollinearity between the 14 variables. The results showed that all the index variance inflation factors (VIF) were lower than five, and only one condition index exceeded 15. The variance ratio of all variables in this dimension is less than 0.9, indicating that there is no collinearity between the independent variables, and it can be used as an independent variable for binary logistic regression.

By constructing a binary logistic regression model for the prognosis of pulpitis, the 14 independent variables of sex, age, root maturation, cave shape, spontaneous pain, nocturnal pain, referred pain, cold test, pain level of cold test, hot test, pain level of hot test, electrical vitality test difference, percussion sensitivity and VPT type met the modeling conditions. The fitting results of the binary logistic regression model constructed with these 14 independent variables are shown in Table 2.

The significance of this model’s Hosmo test is >0.05, which can be considered a good fit. It can be seen from Table 2 that the significance of the 14 independent variables is greater than 0.05, so, according to the *p* = 0.05 level, the above factors are not yet shown to have a significant impact on the prognosis of patients.

## 4. Discussion

Dental caries are very common in both teenagers and adults, and pulp exposure due to caries also frequently occurs in clinical practice [15]. In the past, due to material limitations, teeth with irreversible pulpitis caused by caries were commonly considered to be a condition that could not be reversed by treatment, regardless of the presence or absence of symptoms of inflammation. Therefore, RCT was the standard therapy to remove the pulp completely. However, with the development of materials, VPT is increasingly applied as a personalized treatment to preserve the pulp and could achieve a good prognosis [2] even when the pulp is diagnosed as irreversible pulpitis.

There are four types of VPT: indirect pulp capping (IPC), DPC, PP and FP. Except for IPC, pulp status can be assessed by direct vision through the perforating hole in the other three types of VPT. DPC, PP and FP applications in irreversible pulpitis were studied. For instance, Suhag et al. found that DPC with MTA could achieve a high success rate (93%) and maintain a stable state within one year [17]. Permanent teeth with signs and symptoms that were indicative of irreversible pulpitis in 6- to18-year-old patients were successfully treated with PP using MTA or Biodentine [18]. Asgary et al. conducted a randomized controlled study on the clinical diagnosis of irreversible pulpitis using a calcium-enriched mixture (CEM) or MTA for FP. This was compared with RCT in a 5-year follow-up, and the success rates of the two treatment methods were comparable [19]. In addition, the results of VPT for teeth with signs and symptoms of irreversible pulpitis, as recorded in systematic reviews, were comparable to those applied to teeth with reversible pulpitis [16,20]. Our study showed DPC, PP and FP could all achieve successful and comparable prognosis when the removal of pulp was determined by the hemostasis time.

Whether the clinician could accurately determine the state and amount of pulp removed during VPT are key factors affecting the prognosis. Although hemostatic time was applied to assess the pulp state, there is no agreement on the hemostasis method and specific time. According to Whitherspoon et al., the inflammatory state of the pulp was considered reversible when the hemostatic time was controlled within 10 min by irrigation with 1% NaOCl [21]. In addition, Qudeimat et al. reported that the VPT treatment was successful for teeth with irreversible pulpitis and a hemostatic time up to even 24 min [22]. Taha et al. used a cotton pellet moistened with 1% NaOCl to contact the exposed pulp for hemostasis before FF directly and achieved a high success rate (100%) [23]. In our study, a more stringent criterion was applied: only teeth with pulp tissue that could be hemostatic within 5 min by direct contact with a cotton pellet moistened with 1% NaOCl were included, and the application of VPT in these cases achieved a high success rate (91.5%), indicating that the hemostatic test was a good clinical index for pulp status. However, not enough data showed the optimal hemostatic time. However, Taha et al. showed no correlation between the time required for hemostasis and the clinical and radiographic outcome [24]. Another study showed additional information to the previous one allowing the conclusion that radiographic and histological outcomes were not affected by hemostasis time in an animal model [25]. The correlation between the time required for hemostasis and pulp state should be further studied.

Age is also a controversial issue in VPT case selection. It was suggested that pulp in elderly patients had more fibers, less stem-cell content and a reduced ability to repair the damage, while the abundant blood supply and defense mechanisms in the pulp of young permanent teeth could improve the VPT success rate [26]. However, it was also reported that age had no effect on DPC prognosis in permanent teeth with carious exposure [27]. In this study, the results showed that age had no significant effect on VPT prognosis using iRoot BP Plus. This may be related to the limited age range in this study. The cases with a wider age range should be included in the subsequent study.

At present, there is no standard guideline for VPT indications, so the preoperative evaluation is particularly important. The diagnosis of irreversible pulpitis relies on the patient’s clinical symptoms and reaction to the temperature test [28]. Spontaneous pain, nocturnal pain and referred pain are typical symptoms of irreversible pulpitis and can reflect pulp status to some extent [29]. In this study, we found that the presence or absence of spontaneous pain, nocturnal pain and referred pain in patients had no significant effect on the prognosis of VPT, which was similar to the findings of Kundzina et al. [30] and Liu et al. [27]. However, due to the limited number of cases in this study, how preoperative symptoms affect the VPT prognosis requires more in-depth study.

Some studies suggested that the cold test is the most effective test for identifying necrotic pulp, so cold testing is still essential before any treatment of teeth with deep caries or pulp-exposed lesions [31]. When left, the pulp exposure caused by caries eventually leads to the progressive spread of pulp inflammation, but pulp sensibility testing might not necessarily reflect this progression [32]. The present study found that neither the results of the cold test and heat test nor the pain index was significantly associated with VPT prognosis.

It is believed that the value of the pulp electrical vitality test could not accurately reflect the pulp status and could only reflect whether Aδ fibers were still able to function normally [32,33]. In this study, the numerical difference between the affected teeth and the control teeth was used as a variable, and no significant effect was found on the prognosis of VPT, regardless of whether the difference in the electrical activity test was greater than 10.

Therefore, we believe that the current diagnostic criteria for irreversible pulpitis are actually based on the empirical speculation of clinical symptoms and signs. In our study, the pulp that was clinically diagnosed with irreversible pulpitis could still be reversed using the VPT technique under strict aseptic conditions; therefore, the traditional diagnostic criteria need to be revised [34].

As another common method of oral physical examination, percussion results could reflect the depth of progression of endodontic inflammation and periapical conditions to some extent. It was also reported that the generation of percussion pain preceded the necrosis of endodontic tissue [35,36]. Additionally, a one-year prospective study showed that the percussion results of teeth with irreversible pulpitis had no significant effect on the prognosis of their crown pulp [23], so patients with positive percussion results were still included in this study. The results showed that percussion results had no significant effect on the prognosis of VPT. Therefore, positive percussion cannot be used as a contraindication for VPT.

The choice of pulp-capping materials is also crucial to the prognosis of VPT. Although MTA is widely used in VPT, it has certain defects as a pulp capping material. MTA needs to be adjusted with normal saline right before use, making the clinical operation steps more cumbersome. In addition, the Bi2O3 contained in MTA may be toxic to dental pulp cells and cause discoloration in the teeth [37]. The iRoot BP Plus used in this study is a new type of premixed material with good biocompatibility and physicochemical properties. Compared with MTA, it does not require mixing and increases clinical convenience [38]. Some scholars studied the therapeutic effect of iRoot BP Plus when applied to mature permanent teeth, with the pulp exposure caused by caries as a pulp-capping agent, and found that a success rate of 98%, 89% and 81% could be obtained at 1, 2 and 3 years or more after surgery, respectively [27]. In the present study, three types of VPT, using iRoot BP Plus as a pulp-capping agent, could all achieve a high success rate, indicating that iRoot BP Plus could be applied in VPT to treat young permanent teeth with carious exposure.

Several limitations are acknowledged for this study. This study tried to offer a new understanding and provide novel thoughts into VPT in irreversible pulpitis; therefore, there was a risk of bias, as only one product was tested, and all participants and personnel were aware of the study and tested materials. This bias could also be caused by the distinctions between the three operators, although there is no statistical difference between the success rate achieved by the three operators. Furthermore, the radiographs could not be standardized, and assessing root development with intraoral radiographs is not accurate. However, this is still the easiest to implement and most common in the clinic, so it would be useful to apply intraoral radiographs to assess the prognosis of VPT in irreversible pulpitis. We had no pictures of the definitive restoration, and no patients older than 20 years old were analyzed. This could be improved in further studies.

## 5. Conclusions

Permanent teeth with irreversible pulpitis caused by caries in 6- to 20-year-old patients were successfully treated with VPT using iRoot BP Plus, including DPC, PP and FP.

## Figures and Tables

**Table 1:** Variable assignment of binary logistic regression model in irreversible pulpitis.

Variable Name	Variable Description	Assignment	Variable Name	Variable Description	Assignment
Sex	Male Female	1 2	Age	[5,10] [10,15] [15,16]	1 2 3
Root maturation	Open Closed	0 1	Cave shape	Occlusal Surface Only Proximal Surface Involved	1 2
Spontaneous pain	No Yes	0 1	Nocturnal pain	No Yes	0 1
Referred pain	No Yes	0 1	Cold test	Tenderness without delaying pain Tenderness with delaying pain <30 s Tenderness with delaying pain ≥30 s	0 1 2
Pain level of cold test	<5 ≥5	0 1	Heat test	Tenderness without delaying pain Tenderness with delaying pain <30 s Tenderness with delaying pain ≥30 s Normal	0 1 2 3
Pain level of heat test	<5 ≥5	0 1	Electrical vitality test difference	<10 ≥10	0 1
Percussion sensitivity	(−) (+)	0 1	VPT type	Partial pulpotomy Full pulpotomy Direct pulp capping	1 2 3

**Table 2:** Results of regression model fitting.

Influencing Factors	B	S.E.	Waild	Sig.	Exp (B)
Sex (1)	1.172	2.186	0.288	0.592	3.228
Age	−4.084	2.919	1.958	0.162	0.017
Root maturation (1)	5.588	4.915	1.293	0.256	3.326
Cave shape (1)	−0.536	2.325	0.053	0.818	0.585
Spontaneous pain (1)	−4.958	1.956	0.000	0.999	0.000
Nocturnal pain (1)	−2.260	1.837	1.515	0.218	0.104
Referred pain (1)	−0.891	2.515	0.125	0.723	0.410
Cold test	2.134	2.972	0.516	0.473	3.446
Pain level of cold test	2.020	2.253	0.804	0.370	3.540
Heat test	0.411	0.659	0.389	0.533	1.508
Pain level of heat test	9.817	1.036	0.000	0.999	4.826
Electrical vitality test difference	3.205	1.886	0.000	0.998	1.121
Percussion sensitivity (1)	−6.208	1.714	2.794	0.095	0.002
VPT type			1.980	0.372	
VPT type (1)	−2.097	2.793	0.564	0.453	0.123
VPT type (2)	0.797	2.382	0.112	0.738	2.219

## Data Availability

The datasets generated and/or analyzed during the current study are not publicly available due individual privacy can be compromised but are available from the corresponding author on reasonable request.
